# Association between kidney function and genetic polymorphisms in atherosclerotic and chronic kidney diseases: A cross-sectional study in Japanese male workers

**DOI:** 10.1371/journal.pone.0185476

**Published:** 2017-10-10

**Authors:** Yoko Kubo, Takahiro Imaizumi, Masahiko Ando, Masahiro Nakatochi, Yoshinari Yasuda, Hiroyuki Honda, Yachiyo Kuwatsuka, Sawako Kato, Kyoko Kikuchi, Takaaki Kondo, Masamitsu Iwata, Toru Nakashima, Hiroshi Yasui, Hideki Takamatsu, Hiroshi Okajima, Yasuko Yoshida, Shoichi Maruyama

**Affiliations:** 1 Center for Advanced Medicine and Clinical Research, Nagoya University Hospital, Nagoya, Japan; 2 Department of Nephrology, Nagoya University Graduate School of Medicine, Nagoya, Japan; 3 Department of CKD Initiatives, Nagoya University Graduate School of Medicine, Nagoya, Japan; 4 Department of Biotechnology, Graduate School of Engineering, Nagoya University, Nagoya, Japan; 5 Program in Radiological and Medical Laboratory Sciences, Nagoya University Graduate School of Medicine, Nagoya, Japan; 6 Safety and Health Promotion Division, Toyota Motor Corporation, Toyota, Japan; 7 R & D Management Division, Toyota Motor Corporation, Toyota, Japan; 8 Innovative Research Center for Preventative Medical Engineering, Nagoya University, Nagoya, Japan; The University of Tokyo, JAPAN

## Abstract

**Background:**

Several single nucleotide polymorphisms (SNPs) have been implicated in the predisposition to chronic kidney disease (CKD). Atherosclerotic disease is deeply involved in the incidence of CKD; however, whether SNPs related to arteriosclerosis are involved in CKD remains unclear. This study aimed to identify SNPs associated with CKD and to examine whether risk allele accumulation is associated with CKD.

**Methods:**

We conducted a cross-sectional study using data of 4814 male workers to examine the association between estimated glomerular filtration rate (eGFR) and 59 candidate polymorphisms (17 CKD, 42 atherosclerotic diseases). We defined the genetic risk score (GRS) as the total number of risk alleles that showed a significant association in this analysis and examined the relationship with CKD (eGFR < 60 ml/min/1.73m^2^). Multivariate logistic regression, discrimination by area under the receiver operating characteristic curve, integrated discrimination improvement (IDI), and category-free net reclassification improvement (cNRI) were evaluated.

**Results:**

In total, 432 participants were categorized as having CKD. We found eight candidate SNPs with *P* value < 0.05 (*CX3CR1* rs3732379, *SHROOM3* rs17319721, *MTP* rs1800591, *PIP5K1B* rs4744712, *APOA5* rs662799, *BRAP* rs3782886, *SPATA5L1* rs2467853, and *MCP1* rs1024611) in the multivariate linear regression adjusted for age, body mass index, systolic blood pressure, and fasting blood glucose. Among these eight SNPs, *BRAP* rs3782886 and *SPATA5L1* rs2467853 were significantly associated with eGFR (false discovery rate < 0.05). GRS was significantly associated with CKD (odds ratio, 1.17; 95% confidence interval, 1.09–1.26). C-statisics improved from 0.775 to 0.780 but showed no statistical significance. However, adding GRS significantly improved IDI and cNRI (0.0057, P = 0.0028, and 0.212, P < 0.001, respectively).

**Conclusions:**

After adjustment for clinical factors, kidney function was associated with *BRAP* rs3782886 and *SPATA5L1* rs2467853 and the GRS for CKD that we developed was associated CKD.

## Introduction

Chronic kidney disease (CKD) is an independent risk factor for end-stage renal disease (ESRD), cardiovascular disease (CVD), and all-cause mortality in the general population [1–4]. The number of patients with CKD is approximately 13.3 million, representing about 13% of the Japanese adult population [5]. The incidence of CKD stage III or higher increases with age [6]. Moreover, lifestyle-related chronic diseases mainly develop in middle age; thus, predicting the risk of kidney disease in this age group is vital to help prevent the progress towards ESRD and CVD.

Several population-based cohort studies have identified clinical factors associated with CKD, including age, hypertension, diabetes mellitus, and smoking [6,7]. These are also common risk factors for developing atherosclerotic diseases [8–11]. While there are numerous clinical risk factors for CKD, genetic factors also contribute to the development of CKD. Based on genome-wide association studies (GWAS) and candidate gene studies, various single nucleotide polymorphisms (SNPs) have been associated with CKD [12–17]. CKD and atherosclerotic diseases have common risk factors; hence, the genetic background may also be common.

We have reported a number of studies that examined several SNPs associated with chronic lifestyle diseases, such as hypertension, obesity, and metabolic syndrome, among medical checkup examinees [18–20]. We developed an SNPs measuring system to evaluate the risk for chronic lifestyle diseases, which includes SNPs related to CKD and atherosclerotic diseases, such as hypertension, stroke, coronary artery disease, and carotid artery disease. In this study, we used the same SNPs and examined their association with kidney function.

This study aimed to evaluate the role of SNPs related to CKD and atherosclerotic diseases in estimated glomerular filtration rate (eGFR) among Japanese male workers and to determine whether the total number of risk alleles is associated with CKD. The results can be useful to predict CKD risk among middle-aged men with or without atherosclerotic risk factors based on the number of genetic risk alleles.

## Materials and methods

### Study participants

All male employees of Toyota Motor Co., Ltd. in Japan and those who participated in the annual health examination in 2010 were enrolled in this study. Only those who consented to DNA sampling were included, and the DNA samples were obtained between 2011 and 2014 from 5112 individuals. In this analysis, we excluded those whose creatinine level data were missing. Consequently, the final number of study participants was 4814. The study protocol was approved by the ethics committee of Nagoya University School of Medicine (No. 1089–4), and the study was conducted in accordance with the guidelines of the Declaration of Helsinki. All the participants provided written informed consent. This trial was registered at umin.ac.jp as UMIN000016266.

### Lifestyle evaluation

The lifestyle of participants was evaluated using self-administered questionnaires, which included items on smoking, stress, exercise habits, and drinking habits. Smoking was defined as the current smoking status of the participants and stress as self-reported psychological stress. Responses for drinking habits were divided into four categories: none, <2 days/week, 3–6 days/ week, and every day. Exercise habits were also divided into three categories: none, <6 days/week, and every day.

### Clinical parameters and outcome definition

The health examinations included physical measurements and serum biochemical measurements. Body mass index (BMI) was calculated as follows: weight (kg)/height (m^2^), while blood pressure (BP) was measured in the sitting position using an automatic sphygmomanometer (Kentaro HBP-9020; Omron, Tokyo, Japan).

The biochemical measurements included total cholesterol, triglyceride (TG), high-density lipoprotein (HDL), low-density lipoprotein (LDL), fasting blood glucose (FBG), HbA1c, uric acid (UA), and creatinine levels. The following equation was employed to calculate eGFR, as described by Matsuo et al. [21]:

$$\text{Estimated}\;\text{GFR}\;\left(\text{eGFR}\right)=194\;\times {\;\text{standardized}\;\text{serum}\;\text{creatinine}}^{-1.094}\times {\;\text{age}}^{-0.287}$$

Moreover, the positive result in the urine dipstick test represents urinary protein. CKD was defined as eGFR < 60 ml/min/m^2^.

### SNP selection

In our previous study, we developed an SNP measuring system including 99 candidate SNPs that were associated with coronary heart disease, hypertension, dyslipidemia, diabetes mellitus, hyperuricemia, renal disease, or obesity [18]. Here, we selected 59 candidate SNPs that were associated with CKD and diseases related to atherosclerosis, including hypertension, coronary artery disease, stroke, and carotid artery disease. Detailed information of SNPs is shown in S1 Table.

### Genotyping the SNPs

DNA was anonymously extracted from blood samples of the participants (0.2 mL each) at DNA Chip Research Inc. (QIAamp^®^ series; QIAGEN K.K., Tokyo, Japan). All SNPs were genotyped using the DigiTag2 assay [22].

Primers, probe sequences, and polymerase chain reaction conditions for the SNPs in the original cohort were similar to those described previously [18]. All the laboratory technicians were blinded to the participants’ identity, demographic characteristics, and study outcomes. Our study’s laboratory protocol was registered at http://dx.doi.org/10.17504/protocols.io.jmick4e

### Statistical analysis

The clinical characteristics of the participants are presented as numbers and percentages for qualitative variables (i.e., smoking, exercise habits, and drinking habits), and as means and standard deviations or medians and interquartile ranges (IQRs) for quantitative variables (i.e., age, BMI, abdominal circumference, systolic blood pressure (SBP), and diastolic blood pressure (DBP)) as appropriate.

For each SNP, a score of 0, 1, or 2 was assigned depending on the number of minor alleles. For example, if A is the minor allele, the genotype aa corresponds to zero minor alleles, Aa corresponds to the presence of one minor allele, and AA corresponds to the presence of two minor alleles. The SNP score was treated as a continuous variable.

Initially, we examined the association between eGFR and SNPs using linear regression, assuming an additive genetic model adjusted for age, BMI, SBP, and FBG. Considering the multiple comparisons of genotypes with eGFR, the false discovery rate (FDR) was calculated from the distribution of *P* values for regression analysis of the 59 polymorphisms. We considered polymorphisms with an FDR of < 0.05 to be strongly associated with eGFR, while polymorphisms with a *P* value < 0.05 were considered to be potentially associated with kidney function. The genetic risk score (GRS) was calculated as the sum of the total number of risk alleles. In addition, we also constructed a weighted model to reflect the difference in the effect of each risk variant. To estimate the effect size of each risk variant, we performed logistic regression analysis in which the dependent variable was the presence of CKD and the independent variables were eight SNPs. The weighted genetic risk score (wGRS) was appropriately calculated based on the estimated weights in the logistic regression analysis. Moreover, based on the logistic regression analysis, we appropriately assigned integer coefficients to each variant to construct the wGRS.

Subsequently, we evaluated the association between CKD and GRS. Multivariate logistic regression analysis adjusted for the factors that we obtained from health examination data and is associated adjusted for the factors obtained from the health examination data revealed an association with CKD. In the multivariable logistic regression analysis, we set three adjusting models as follows:

Model 1: adjusted for age, BMI, SBP, and FBG
Model 2: model 1 + exercise habit, drinking habit, smoking, and stress
Model 3: model 2 + LDL cholesterol, uric acid, and urinary protein through positive urine dipstick test

Model 1 contains major metabolic components and has a set of variables common in the multiple regression analysis of eGFR. Model 2 contains lifestyle information, which we selected based on literature review [23,24]. Model 3 contains other blood chemical tests, including urine dipstick test. Constructing multiple models enables the evaluation of whether lifestyle and other factors (e.g., proteinuria or serum uric acid) could affect GRS.

We also evaluated c-statistics, integrated discrimination improvement (IDI), and net reclassification improvement (NRI) to determine how the GRS improved outcome prediction [25,26]. IDI and NRI were assessed as category-free and thus could represent the average improvement in sensitivity across all possible cutoffs. *P* < 0.05 was considered statistically significant. The data were analyzed using Stata MP 14.2 (StataCorp LLC, Texas, USA).

## Results

### Clinical characteristics

Clinical characteristics (clinical parameters, lifestyle data, and meal purchase data) of the study participants are shown in Table 1. The number of the participants with CKD was 432 (9.85%). Participants with CKD were older and had higher BMI, SBP and DBP, BUN, UA, TG, LDL cholesterol, and FBG. The proportion of current smokers and daily drinkers was higher in the control group.

**Table 1:** Clinical characteristics between the participants with or without chronic kidney disease.

Characteristics	Number of data	Total	CKD (N = 432)	Control (N = 4382)	*P* value
**Age**	4814	47.3±6.25	50.2±4.76	47.0±6.30	<0.001*
**Body mass index**	4814	23.7±3.44	24.7±3.50	23.6±3.42	<0.001*
**Systolic blood pressure (mmHg)**	4814	121.2±13.8	123.6±14.0	121.0±13.7	<0.001*
**Diastolic blood pressure (mmHg)**	4810	76.9±10.1	79.7±9.74	76.6±10.1	<0.001*
**Blood urea nitrogen (mg/dL)**	4814	14.1±3.62	16.6±5.61	13.9±3.26	<0.001*
**Creatinine (mg/dL)**	4814	0.8 (0.8–0.9)	1.1 (1.1–1.2)	0.8 (0.8–0.9)	<0.001*
**eGFR (ml/min/1.73m^2^)**	4814	77.6±14.4	52.8±7.84	80.0±12.5	<0.001*
**Uric acid (mg/dL)**	4814	6.08±1.28	6.89±1.36	6.01±1.25	0.039*
**Total cholesterol (mg/dL)**	4814	203.8±33.7	207.0±35.1	203.5±33.5	0.096
**Triglyceride (mg/dL)**	4813	102 (70–153)	111 (79–167.5)	101 (70–150)	<0.001*
**High density lipoprotein (mg/dL)**	4814	58.2±15.1	57.0±16.1	58.3±15.0	0.10
**Low density lipoprotein (mg/dL)**	4814	123.2±30.4	126.5±31.1	122.9±30.3	0.018*
**Fasting blood glucose**	4814	92 (87–99)	94 (88–101)	92 (87–99)	0.0032*
**HbA_1_c**	4814	5.32±0.64	5.34±0.59	5.32±0.64	0.55
**Urinary protein positivity on dipstick**	4814	263, 5.46%	59, 13.66%	204, 4.66%	<0.001*
**Lifestyle**					
** Exercise habits**	4805				<0.001*
** None**		1562, 32.51%	100, 23.26%	1462, 33.42%	
** <6 days per week**		2425, 50.47%	248, 57.67%	2177, 49.76%	
** Every day**		818, 17.02%	82, 19.07%	736, 16.82%	
** Drinking habits**	4806				<0.001*
** None**		1130, 23.51%	113, 26.28%	1017, 23.24%	
** <2 days per week**		1168, 24.3%	123, 28.6%	1045, 23.88%	
** 3–6 days per week**		780, 16.23%	77, 17.91%	703, 16.06%	
** Every day**		1728, 35.96%	117, 27.21%	1611, 36.81%	
** Smoking**	4810	1783, 37.07%	87, 20.19%	1696, 38.73%	<0.001*
** Stress**	4806	3921, 81.59%	339, 78.84%	3582, 81.86%	0.12

CKD, chronic kidney disease. Continuous data are presented as mean ± SD or medians (1st quartile, 3rd quartile) and categorical data as n values (%).

*P value < 0.05.

### Linear regression analysis between SNPs and eGFR

S2 Table showed the univariate and multivariate regression analyses between eGFR and SNPs. In the multivariate linear regression adjusted for age, BMI, SBP, and FBG, we found eight candidate SNPs with *P* value < 0.05: *CX3CR1* rs3732379, *SHROOM3* rs17319721, *MTP* rs1800591, *PIP5K1B* rs4744712, *APOA5* rs662799, *BRAP* rs3782886, *SPATA5L1* rs2467853, and *MCP1* rs1024611. Among these SNPs, *BRAP* rs3782886 and *SPATA5L1* rs2467853 were significantly associated with eGFR (FDR < 0.05) (Table 2).

**Table 2:** Multivariate linear regression analysis of the association between eGFR and SNPs.

rs#	Near gene	Major/minor allele	Risk allele^a^	Coefficient^b^	95%CI	*P* value	FDR
**rs3732379**	*CX3CR1*	C/T	C	1.25	(0.004, 2.50)	0.049	0.36
**rs17319721**	*SHROOM3*	G/A	A	-1.16	(-2.15, -0.16)	0.023	0.24
**rs1800591**	*MTP*	G/T	G	0.99	(0.26, 1.72)	0.0077	0.13
**rs4744712**	*PIP5K1B*	C/A	A	-0.75	(-1.31, -0.19)	0.0088	0.13
**rs662799**	*APOA5*	A/G	G	-0.61	(-1.18, -0.046)	0.034	0.29
**rs3782886**	*BRAP*	A/G	G	-1.63	(-2.25, -1.02)	<0.0001	<0.0001^c^
**rs2467853**	*SPATA5L1*	G/T	G	2.07	(0.89, 3.24)	0.0006	0.018 ^c^
**rs1024611**	*MCP1*	C/T	T	-0.65	(-1.22, -0.084)	0.025	0.24

rs#, rs number; CI, confidence interval; FDR, false discovery rate

^a^ The allele that decreased eGFR was defined as risk allele.

^b^ Coefficient represents the value of eGFR increase as the number of minor allele increased by 1

^c^ FDR < 0.05.

### Effects of genetic risk score on CKD

Linear regression analysis revealed eight candidate SNPs associated with kidney function. Subsequently, we calculated GRS by adding the total number of risk alleles. The mean value of GRS was 8.3 (± 1.6), and the maximum and minimum values were 14 and 3, respectively.

Multiple models analyzing the association between GRS and CKD are shown in Table 3. After adjusting for relevant clinical factors, GRS was shown to be an independent factor associated with CKD. C-statistics showed marginally significant improvement by using GRS (*P* = 0.061), whereas IDI and NRI showed a significant improvement (P = 0.0028 and < 0.0001, respectively).

**Table 3:** Univariate and multivariate logistic regression analysis of SNP Score and CKD.

	OR	95% CI	*P* value	C-statistics with GRS	C-statistics without GRS	*P* value	IDI	*P* value	NRI	*P* value
**Univariate**	1.16	[1.08–1.23]	<0.001*	0.562	-	-	-	-	-	-
**Model 1**	1.17	[1.10–1.25]	<0.001*	0.686	0.674	0.0090*	0.0049	<0.001*	0.203	<0.001*
**Model 2**	1.16	[1.08–1.24]	<0.001*	0.713	0.706	0.062	0.0046	<0.001*	0.200	<0.001*
**Model 3**	1.15	[1.08–1.23]	<0.001*	0.716	0.709	0.061	0.0057	<0.001*	0.212	<0.001*

CI, confidence interval; GRS, genetic risk score; IDI, integrated discrimination improvement; NRI, net reclassification improvement. Model 1: adjusted for age, body mass index, systolic blood pressure, and fasting blood glucose. Model 2: model 1 + exercise habit, drinking habit, smoking, and stress. Model 3: model 2 + LDL cholesterol and fasting blood glucose, uric acid, and urinary protein through positive urine dipstick

* *P* < 0.05

We constructed wGRS based on the logistic regression analysis shown in S3 Table. We multiplied the coefficient from the logistic regression analysis by 20 then added 25. The mean value of wGRS was 29.1 (± 5.7), and the maximum and minimum values were 46 and 1, respectively.

Multiple models analyzing the association between wGRS and CKD are shown in S4 Table. We also showed that wGRS is an independent factor associated with CKD. C-statistics, IDI, and NRI showed statistically significant improvement by using wGRS (*P* = 0.040, < 0.001, < 0.001, respectively).

## Discussion

In this study, we showed a significant association between kidney function and genetic polymorphism, specifically *BRAP* rs3782886 and *SPATA5L1* rs2467853, after adjustment for clinical factors including lifestyle information. Moreover, we developed a GRS for chronic kidney disease, and used it in male workers who regularly undergo health checkups.

Several GWAS and candidate gene studies revealed the SNPs associated with kidney disease or kidney function [12–17]. Our study was unique as annual health examination data, particularly among middle-aged men, in a manufacturing company were used and a genetic risk score was developed. We developed a GRS combining the risk alleles in eight SNPs associated with kidney function, and examined its utility.

*BRAP* rs3782886 and *ALDH2* rs671 have strong linkage disequilibrium according to the HapMap database [27]. Meta-analysis of GWAS in East Asian populations showed associations between the SNP *ALDH2* rs671 and obesity and various cardiovascular risk factors and coronary artery disease (CAD) [28]. The minor allele of *ALDH2* rs671 was associated with CAD, high LDL, and low HDL, while the major allele was associated with high blood pressure, and high FBG. Another meta-analysis of GWAS in East Asian populations showed associations between the SNP *ALDH2* rs671 and kidney function [17] and revealed that the minor allele was associated with worse kidney function. In our study, the minor allele of *BRAP* rs3782886 was associated with lower kidney function, which is consistent with a previous study. A prospective study is necessary to investigate how *BRAP* rs3782886 affects the development of CVD and ESRD.

Moreover, similar to a previous study, our study also showed a significant association between *SPATA5L1* rs2467853 and kidney function [29]. *SPATA5L1* rs2467853 is an intronic SNP associated with glomerular filtration rate, which was estimated using serum creatinine, among European-descent participants from multiple cohorts; however, the biological significance remains unclear. Furthermore, a previous study showed that the *CX3CR1* rs3732379 SNP CC genotype is associated with increased risk of delayed graft function among patients who received renal transplantation [30]. In a study that first reported on the kidney function of a general population, CX3CR1 rs3732379 SNP was associated with atherosclerosis of the coronary artery [31], *SHROOM3* rs17319721 and *PIP5K1B* rs4744712 were associated with kidney disease [29,32]. The minor allele of *MTP* rs1800591 was shown to be associated with hypertension in a Mexican population [33] and, in another study, with lower cholesterol level but ischemic heart disease [34]; in the present study, the major allele of *MTP* rs1800591 was associated with worse kidney function. No related study on kidney function exists; thus, further studies are needed. Moreover, *APOA5* rs662799 was associated with CKD in the Japanese general population [35]. *MCP1* rs1024611 was associated with carotid artery plaque, ischemic stroke (IS), and ischemic heart disease (IHD) [36,37]. The rs1024611C(G) allele is associated with increased serum MCP-1 levels and enhanced leukocyte recruitment to the tissues among Caucasians, which in turn could lead to atherosclerosis [38]. One meta-analysis showed that C(G) allele is a risk allele for IHD and IS [36], while some previous studies showed that T(A) is a risk allele for the development of diabetic nephropathy among the Asian population [39,40]. Whether *MCP1* rs1024611 causes arteriosclerosis via MCP-1 level change among Asians remains unclear; hence, further studies are needed.

Limitations of this study include the possibility of a sampling bias. Participation in the study was based on the willingness of individuals; consequently, the number of study participants was limited. New findings have been accumulated since we started this research; thus, the selection of SNPs could also be a limitation. In addition, two further limitations regarding model construction in this study were noted: First, we used the same sample to get the weight for the weighted genetic score analysis. Thus, the results for weighted models could have been inflated. We could not have a different cohort to validate the effect of GRS. Hence, further studies should perform such an analysis with a different sample. Second, we also considered wGRS to reflect the difference in the effect of each variant. However, a comparison between the simple counting method (using GRS) and the scoring system (using wGRS) showed that AUCs were similar. Therefore, the simple counting of risk alleles could be feasible and useful. Lastly, the cross-sectional nature of this study limits our ability to estimate the longitudinal effects. Long-term observation of our study’s cohort will help in understanding and identifying individuals at a high risk of developing CKD and CVD.

In conclusion, a significant association between kidney function and genetic polymorphism, specifically *BRAP* rs3782886 and *SPATA5L1* rs2467853, exists. The GRS for chronic kidney disease that we developed was found to be useful in risk evaluation among middle-aged Japanese men. Nevertheless, validating the utility of GRS among other populations is necessary. In addition, a prospective observation of high-risk populations is warranted to confirm such association.

## Supporting information

S1 Table Characteristics of genotyped SNPs. (DOCX)

S2 Table Crude and adjusted logistic regression analysis of association between eGFR and SNPs. (DOCX)

S3 Table Result of logistic regression analysis performed for weighting. (DOCX)

S4 Table Univariate and multivariate logistic regression analyses of weighted SNP score and CKD. (DOCX)

S1 Dataset Clinical data of 4814 study participants. (XLSX)
