# Supplementary material for: Association between kidney function and genetic polymorphisms in atherosclerotic and chronic kidney diseases: A cross-sectional study in Japanese male workers
Source: PLoS One. 2017 Oct 10;12(10):e0185476. doi: 10.1371/journal.pone.0185476 (PMC5634546; doi:10.1371/journal.pone.0185476)
Supplement: S1 Table — (DOCX) [file pone.0185476.s001.docx]

**S1 Table** Characteristics of genotyped SNPs

| rs# | Chr | Position (GRCh37) | Near gene | A/a | Genotype | | | HWE  *P* value | Associated diseases / traits | Reference |
| --- | --- | --- | --- | --- | --- | --- | --- | --- | --- | --- |
|  |  |  |  |  | AA | Aa | aa |  |  |  |
| rs1801133 | 1 | 11856378 | *MTHFR* | C/T | 1782 | 2296 | 730 | 0.86 | CKD | Jamison, et al. 2009　[1] |
| rs5063 | 1 | 11907648 | *ANP* | G/A | 3973 | 787 | 48 | 0.19 | stroke | Rubattu, et al. 1999 [2] |
| rs1764391 | 1 | 35260769 | *GJA4* | C/T | 3174 | 1478 | 156 | 0.34 | CAD | Yamada, et al. 2002 [3] |
| rs1137100 | 1 | 66036441 | *LEPR* | G/A | 2981 | 1590 | 237 | 0.18 | atherosclerosis | Saukko, et al. 2010 [4] |
| rs267734 | 1 | 150951477 | *CERS2, ANXA9* | A/G | 4420 | 386 | 4 | 0.19 | CKD | Böger, et al. 2011 [5] |
| rs1417938 | 1 | 159684186 | *CRP* | T/A | 4203 | 593 | 16 | 0.40 | CVD | Lange, et al. 2006 [6] |
| rs6131 | 1 | 169580885 | *SELP* | G/A | 3415 | 1239 | 119 | 0.58 | CAD | Tregouet, et al. 2002　[7] |
| rs1800872 | 1 | 206946407 | *IL10* | T/G | 2204 | 2085 | 517 | 0.47 | CAD | Koch, et al. 2001 [8] |
| rs699 | 1 | 230845794 | *AGT* | C/T | 3143 | 1493 | 171 | 0.74 | hypertension | Li, et al. 2014 [9] |
| rs4762 | 1 | 230845977 | *AGT* | C/T | 3867 | 894 | 50 | 0.94 | CHF | Procopciuc, et al. 2011 [10] |
| rs1805087 | 1 | 237048500 | *MTR* | A/G | 3180 | 1444 | 187 | 0.16 | carotid intima thickness | Sun, et al. 2016 [11] |
| rs1260326 | 2 | 27730940 | *GCKR* | T/C | 1539 | 2348 | 923 | 0.62 | CKD | Böger, et al. 2011 [5] |
| rs2228048 | 3 | 30713842 | *TGFBR2* | C/T | 2712 | 1769 | 324 | 0.13 | CKD | McKnight, et al. 2007 [12] |
| rs933135 | 3 | 38052725 | *PLCD1* | C/T | 2602 | 1868 | 338 | 0.91 | CAD | Nakano, et al. 2002 [13] |
| rs3732379 | 3 | 39307256 | *CX3CR1* | C/T | 4341 | 450 | 13 | 0.65 | CAD | McDermott, et al. 2001 [14] |
| rs1050450 | 3 | 49394834 | *GPX1* | C/T | 4167 | 621 | 17 | 0.26 | hypertension | Yamada, et al. 2007 [15] |
| rs347685 | 3 | 141807137 | *TFDP2* | A/C | 2333 | 2011 | 462 | 0.34 | CKD | Böger, et al. 2011 [5] |
| rs388915 | 3 | 148447756 | *AT2R1* | A/G | 3430 | 1255 | 113 | 0.96 | CKD | Osawa, et al. 2007 [16] |
| rs5186 | 3 | 148459988 | *AT2R1* | A/C | 4073 | 705 | 31 | 0.92 | hypertension | Kainulainen, et al. 1999 [17] |
| rs6141 | 3 | 184090266 | *THPO* | G/A | 1488 | 2348 | 969 | 0.45 | CAD | Webb, et al. 2001 [18] |
| rs4961 | 4 | 2906707 | *ADD1* | T/G | 1448 | 2338 | 1027 | 0.15 | hypertension | Cusi, et al. 1997 [19] |
| rs1014290 | 4 | 10001861 | *SLC2A9* | T/C | 1649 | 2354 | 801 | 0.44 | CKD | Hamajima, et al. 2011 [20] |
| rs17319721 | 4 | 77368847 | *SHROOM3* | G/A | 4054 | 724 | 31 | 0.92 | CKD | Köttgen, et al. 2009 [21] |
| rs1800591 | 4 | 100495488 | *MTP* | G/T | 3359 | 1314 | 135 | 0.64 | hypertension | Ríos-González, et al. 2014 [22] |
| rs1799883 | 4 | 120241902 | *FABP2* | C/T | 2057 | 2156 | 589 | 0.52 | CAD | Vimaleswaran, et al. 2006 [23] |
| rs1801394 | 5 | 7870973 | *MTRR* | A/G | 2331 | 2026 | 443 | 0.92 | CKD | Hishida, et al. 2013 [24] |
| rs11959928 | 5 | 39397132 | *DAB2* | A/T | 2917 | 1643 | 252 | 0.30 | CKD | Böger, et al. 2011 [5] |
| rs2569190 | 5 | 140012916 | *CD14R* | A/G | 1387 | 2412 | 1000 | 0.43 | CAD | Koenig, et al. 2002 [25] |
| rs2070600 | 6 | 32151443 | *AGER* | G/A | 3496 | 1209 | 106 | 0.91 | CAD | Lu, et al. 2011 [26] |
| rs881858 | 6 | 43806609 | *VEGFA* | A/G | 3725 | 1011 | 73 | 0.63 | CKD | Böger, et al. 2011 [5] |
| rs2431260 | 6 | 152192331 | *ESR1* | C/G | 1542 | 2309 | 959 | 0.079 | atherosclerosis | Gallagher, et al. 2007 [27] |
| rs2070744 | 7 | 150690079 | *NOS3* | T/C | 3795 | 963 | 52 | 0.34 | atherosclerosis | Kullo, et al. 2008 [28] |
| rs1799983 | 7 | 150696111 | *NOS3* | G/T | 4096 | 672 | 35 | 0.19 | carotid plaque | Lembo, et al. 2001 [29] |
| rs328 | 8 | 19819724 | *LPL* | C/G | 3678 | 1051 | 76 | 0.90 | CAD | Wittrup, et al. 1999 [30] |
| rs10109414 | 8 | 23751151 | *STC1* | C/T | 3083 | 1529 | 195 | 0.75 | CKD | Böger, et al. 2011 [5] |
| rs1346044 | 8 | 31024654 | *WRN* | T/C | 4088 | 694 | 24 | 0.42 | atherosclerosis | Castro, et al. 2000 [31] |
| rs4744712 | 9 | 71434707 | *PIP5K1B* | C/A | 1823 | 2281 | 705 | 0.85 | CKD | Böger, et al. 2011 [5] |
| rs501120 | 10 | 44753867 | *10q11* | A/G | 2109 | 2141 | 559 | 0.65 | CAD | Samani, et al. 2007 [32] |
| rs662799 | 11 | 116663707 | *APOA5* | A/G | 2069 | 2143 | 595 | 0.28 | CAD | Martinelli, et al. 2007 [33] |
| rs5443 | 12 | 6954875 | *GNB3* | T/C | 1265 | 2335 | 1205 | 0.053 | CAD | Frey, et al. 2014 [34] |
| rs11053646 | 12 | 10313448 | *OLR1, LOX1* | G/C | 3109 | 1486 | 211 | 0.051 | stroke | Au, et al. 2015 [35] |
| rs958812 | 12 | 12049662 | *LRP6* | C/T | 1920 | 2205 | 681 | 0.24 | CAD | Mani, et al. 2007 [36] |
| rs1799986 | 12 | 57535266 | *LRP1* | C/T | 3817 | 925 | 65 | 0.30 | CAD | Pocathikorn, et al. 2003 [37] |
| rs3782886 | 12 | 112110489 | *BRAP* | A/G | 2614 | 1852 | 333 | 0.85 | CAD | Hirokawa, et al. 2015 [38] |
| rs1411766 | 13 | 110252160 | *ch 13q* | C/T | 3811 | 933 | 54 | 0.77 | CKD | Maeda, et al. 2010 [39] |
| rs6046 | 13 | 113773159 | *F7* | C/T | 4212 | 581 | 18 | 0.81 | CAD | Shimokata, et al. 2002 [40] |
| rs2467853 | 15 | 45698793 | *SPATA5L1* | G/T | 4261 | 508 | 16 | 0.79 | CKD | Köttgen, et al. 2009 [21] |
| rs1800588 | 15 | 58723675 | *LIPC* | C/T | 1251 | 2419 | 1143 | 0.71 | CAD | Andersen, et al. 2003 [41] |
| rs6495446 | 15 | 80154982 | *MTHFS* | C/T | 3333 | 1327 | 146 | 0.33 | CHF | Kottgen, et al. 2008 [42] |
| rs5882 | 16 | 57016092 | *CETP* | G/A | 1273 | 2370 | 1166 | 0.34 | CAD | Blankenberg, et al. 2003 [43] |
| rs4673 | 16 | 88713236 | *CYBA* | C/T | 3910 | 830 | 53 | 0.22 | CAD | Cahilly, et al. 2000 [44] |
| rs1024611 | 17 | 32579788 | *MCP1* | C/T | 2062 | 2151 | 590 | 0.43 | carotid plaque | Brenner, et al. 2006 [45] |
| rs2333227 | 17 | 56358762 | *MPO* | C/T | 3894 | 869 | 44 | 0.63 | stroke | Hoy, et al. 2003 [46] |
| rs5498 | 19 | 10395683 | *ICAM1* | A/G | 1765 | 2294 | 723 | 0.63 | stroke | Pola, et al. 2003 [47] |
| rs1800469 | 19 | 41860296 | *TGFB1* | T/C | 1271 | 2405 | 1129 | 0.91 | CAD | Crobu, et al. 2008 [48] |
| rs405509 | 19 | 45408836 | *APOE* | A/C | 2451 | 1952 | 402 | 0.65 | CKD | Yoshida, et al. 2009 [49] |
| rs13038305 | 20 | 23610262 | *CST3-CST9* | G/A | 3667 | 1049 | 94 | 0.071 | CKD | Köttgen, et al. 2009 [21] |
| rs3918242 | 20 | 44635976 | *MMP9* | C/T | 3385 | 1316 | 112 | 0.26 | CAD | Zhang, et al. 1999 [50] |
| rs5629 | 20 | 48129706 | *PTGIS* | C/A | 2776 | 1766 | 266 | 0.53 | hypertension | Yamada, et al. 2006 [51] |

rs#, rs number; Chr, chromosome; A, major allele; a, minor allele; HWE, Hardy Weinberg equilibrium; CVD, cardiovascular disease; CAD, coronary artery disease; CKD, chronic kidney disease; CHF, congestive heart failure

HWE P values were calculated by Fisher’s exact test.

[reference list]

1. Jamison RL, Shih M-C, Humphries DE, Guarino PD, Kaufman JS, Goldfarb DS, et al. Effect of the MTHFR C677T and A1298C polymorphisms on survival in patients with advanced CKD and ESRD: a prospective study. Am J Kidney Dis. 2009;53: 779–89.

2. Rubattu S, Ridker P, Stampfer MJ, Volpe M, Hennekens CH, Lindpaintner K. The gene encoding atrial natriuretic peptide and the risk of human stroke. Circulation. 1999;100: 1722–6.

3. Yamada Y, Izawa H, Ichihara S, Takatsu F, Ishihara H, Hirayama H, et al. Prediction of the risk of myocardial infarction from polymorphisms in candidate genes. N Engl J Med. 2002;347: 1916–23.

4. Saukko M, Kesäniemi YA, Ukkola O. Leptin receptor Lys109Arg and Gln223Arg polymorphisms are associated with early atherosclerosis. Metab Syndr Relat Disord. 2010;8: 425–30.

5. Böger CA, Gorski M, Li M, Hoffmann MM, Huang C, Yang Q, et al. Association of eGFR-Related Loci Identified by GWAS with Incident CKD and ESRD. Kim SK, editor. PLoS Genet. 2011;7: e1002292.

6. Lange LA, Carlson CS, Hindorff LA, Lange EM, Walston J, Durda JP, et al. Association of polymorphisms in the CRP gene with circulating C-reactive protein levels and cardiovascular events. JAMA. 2006;296: 2703–11.

7. Tregouet D-A, Barbaux S, Escolano S, Tahri N, Golmard J-L, Tiret L, et al. Specific haplotypes of the P-selectin gene are associated with myocardial infarction. Hum Mol Genet. 2002;11: 2015–23.

8. Koch W, Kastrati A, Böttiger C, Mehilli J, von Beckerath N, Schömig A. Interleukin-10 and tumor necrosis factor gene polymorphisms and risk of coronary artery disease and myocardial infarction. Atherosclerosis. 2001;159: 137–44.

9. Li H, Du Z, Zhang L, Wu T, Deng Z, Li J, et al. The relationship between angiotensinogen gene polymorphisms and essential hypertension in a Northern Han Chinese population. Angiology. 2014;65: 614–9.

10. Procopciuc LM, Caracostea G, Zaharie G, Puscas M, Iordache G, Popa M, et al. Maternal/newborn genotype contribution of the renin-angiotensin system (Met235Thr, Thr174Met, I/D-ACE, A2350G-ACE, A1166C-AT2R1, C3123A- AT2R2, 83A/G-REN) to the risk of pre-eclampsia: a Romanian study. J Renin Angiotensin Aldosterone Syst. 2011;12: 539–48.

11. Sun K, Song J, Liu K, Fang K, Wang L, Wang X, et al. Associations between homocysteine metabolism related SNPs and carotid intima-media thickness: a Chinese sib pair study. J Thromb Thrombolysis. 2016; doi:10.1007/s11239-016-1449-x

12. McKnight AJ, Savage DA, Patterson CC, Sadlier D, Maxwell AP. Resequencing of genes for transforming growth factor beta1 (TGFB1) type 1 and 2 receptors (TGFBR1, TGFBR2), and association analysis of variants with diabetic nephropathy. BMC Med Genet. 2007;8: 5.

13. Nakano T, Osanai T, Tomita H, Sekimata M, Homma Y, Okumura K. Enhanced activity of variant phospholipase C-delta1 protein (R257H) detected in patients with coronary artery spasm. Circulation. 2002;105: 2024–9.

14. McDermott DH, Halcox JP, Schenke WH, Waclawiw MA, Merrell MN, Epstein N, et al. Association between polymorphism in the chemokine receptor CX3CR1 and coronary vascular endothelial dysfunction and atherosclerosis. Circ Res. 2001;89: 401–7.

15. Yamada Y, Ando F, Shimokata H. Association of gene polymorphisms with blood pressure and the prevalence of hypertension in community-dwelling Japanese individuals. Int J Mol Med. 2007;19: 675–83.

16. Osawa N, Koya D, Araki S, Uzu T, Tsunoda T, Kashiwagi A, et al. Combinational effect of genes for the renin-angiotensin system in conferring susceptibility to diabetic nephropathy. J Hum Genet. 2007;52: 143–51.

17. Kainulainen K, Perola M, Terwilliger J, Kaprio J, Koskenvuo M, Syvänena C, et al. Evidence for involvement of the type 1 angiotensin II receptor locus in essential hypertension. Hypertension. 1999;33: 844–9.

18. Webb KE, Martin JF, Hamsten A, Eriksson P, Iacoviello L, Gattone M, et al. Polymorphisms in the thrombopoietin gene are associated with risk of myocardial infarction at a young age. Atherosclerosis. 2001;154: 703–11.

19. Cusi D, Barlassina C, Azzani T, Casari G, Citterio L, Devoto M, et al. Polymorphisms of alpha-adducin and salt sensitivity in patients with essential hypertension. Lancet (London, England). 1997;349: 1353–7.

20. Hamajima N, Okada R, Kawai S, Hishida A, Morita E, Yin G, et al. Significant association of serum uric acid levels with SLC2A9 rs11722228 among a Japanese population. Mol Genet Metab. 2011;103: 378–82.

21. Köttgen A, Glazer NL, Dehghan A, Hwang S-J, Katz R, Li M, et al. Multiple loci associated with indices of renal function and chronic kidney disease. Nat Genet. 2009;41: 712–7.

22. Ríos-González BE, Ibarra-Cortés B, Ramírez-López G, Sánchez-Corona J, Magaña-Torres MT. Association of polymorphisms of genes involved in lipid metabolism with blood pressure and lipid values in mexican hypertensive individuals. Dis Markers. 2014;2014: 150358.

23. Vimaleswaran KS, Radha V, Mohan V. Thr54 allele carriers of the Ala54Thr variant of FABP2 gene have associations with metabolic syndrome and hypertriglyceridemia in urban South Indians. Metabolism. 2006;55: 1222–6.

24. Hishida A, Okada R, Guang Y, Naito M, Wakai K, Hosono S, et al. MTHFR, MTR and MTRR polymorphisms and risk of chronic kidney disease in Japanese: cross-sectional data from the J-MICC Study. Int Urol Nephrol. 2013;45: 1613–20.

25. Koenig W, Khuseyinova N, Hoffmann MM, März W, Fröhlich M, Hoffmeister A, et al. CD14 C(-260)-->T polymorphism, plasma levels of the soluble endotoxin receptor CD14, their association with chronic infections and risk of stable coronary artery disease. J Am Coll Cardiol. 2002;40: 34–42.

26. Lu W, Feng B, Xie G, Liu F. Association of AGER gene G82S polymorphism with the severity of coronary artery disease in Chinese Han population. Clin Endocrinol (Oxf). 2011;75: 470–4.

27. Gallagher CJ, Langefeld CD, Gordon CJ, Campbell JK, Mychaleckyj JC, Mychalecky JC, et al. Association of the estrogen receptor-alpha gene with the metabolic syndrome and its component traits in African-American families: the Insulin Resistance Atherosclerosis Family Study. Diabetes. 2007;56: 2135–41.

28. Kullo IJ, Greene MT, Boerwinkle E, Chu J, Turner ST, Kardia SLR. Association of polymorphisms in NOS3 with the ankle-brachial index in hypertensive adults. Atherosclerosis. 2008;196: 905–912.

29. Lembo G, De Luca N, Battagli C, Iovino G, Aretini A, Musicco M, et al. A common variant of endothelial nitric oxide synthase (Glu298Asp) is an independent risk factor for carotid atherosclerosis. Stroke. 2001;32: 735–40.

30. Wittrup HH, Tybjaerg-Hansen A, Nordestgaard BG. Lipoprotein lipase mutations, plasma lipids and lipoproteins, and risk of ischemic heart disease. A meta-analysis. Circulation. 1999;99: 2901–7.

31. Castro E, Edland SD, Lee L, Ogburn CE, Deeb SS, Brown G, et al. Polymorphisms at the Werner locus: II. 1074Leu/Phe, 1367Cys/Arg, longevity, and atherosclerosis. Am J Med Genet. 2000;95: 374–80.

32. Samani NJ, Erdmann J, Hall AS, Hengstenberg C, Mangino M, Mayer B, et al. Genomewide association analysis of coronary artery disease. N Engl J Med. 2007;357: 443–53.

33. Martinelli N, Trabetti E, Bassi A, Girelli D, Friso S, Pizzolo F, et al. The -1131 T&gt;C and S19W APOA5 gene polymorphisms are associated with high levels of triglycerides and apolipoprotein C-III, but not with coronary artery disease: an angiographic study. Atherosclerosis. 2007;191: 409–17.

34. Frey UH, Moebus S, Möhlenkamp S, Kälsch H, Bauer M, Lehmann N, et al. GNB3 gene 825 TT variant predicts hard coronary events in the population-based Heinz Nixdorf Recall study. Atherosclerosis. 2014;237: 437–42.

35. Au A, Griffiths LR, Cheng K-K, Wee Kooi C, Irene L, Keat Wei L. The Influence of OLR1 and PCSK9 Gene Polymorphisms on Ischemic Stroke: Evidence from a Meta-Analysis. Sci Rep. 2015;5: 18224.

36. Mani A, Radhakrishnan J, Wang H, Mani A, Mani M-A, Nelson-Williams C, et al. LRP6 mutation in a family with early coronary disease and metabolic risk factors. Science. 2007;315: 1278–82.

37. Pocathikorn A, Granath B, Thiry E, Van Leuven F, Taylor R, Mamotte C. Influence of exonic polymorphisms in the gene for LDL receptor-related protein (LRP) on risk of coronary artery disease. Atherosclerosis. 2003;168: 115–21.

38. Hirokawa M, Morita H, Tajima T, Takahashi A, Ashikawa K, Miya F, et al. A genome-wide association study identifies PLCL2 and AP3D1-DOT1L-SF3A2 as new susceptibility loci for myocardial infarction in Japanese. Eur J Hum Genet. 2015;23: 374–80.

39. Maeda S, Araki S-I, Babazono T, Toyoda M, Umezono T, Kawai K, et al. Replication study for the association between four Loci identified by a genome-wide association study on European American subjects with type 1 diabetes and susceptibility to diabetic nephropathy in Japanese subjects with type 2 diabetes. Diabetes. 2010;59: 2075–9.

40. Shimokata K, Kondo T, Ohno M, Takeshita K, Inden Y, Iino S, et al. Effects of coagulation Factor VII polymorphisms on the coronary artery disease in Japanese: Factor VII polymorphism and coronary disease. Thromb Res. 2002;105: 493–8.

41. Andersen R V, Wittrup HH, Tybjaerg-Hansen A, Steffensen R, Schnohr P, Nordestgaard BG. Hepatic lipase mutations,elevated high-density lipoprotein cholesterol, and increased risk of ischemic heart disease: the Copenhagen City Heart Study. J Am Coll Cardiol. 2003;41: 1972–82.

42. Kottgen A, Kao WHL, Hwang S-J, Boerwinkle E, Yang Q, Levy D, et al. Genome-wide association study for renal traits in the Framingham Heart and Atherosclerosis Risk in Communities Studies. BMC Med Genet. 2008;9: 49.

43. Blankenberg S, Rupprecht HJ, Bickel C, Jiang X-C, Poirier O, Lackner KJ, et al. Common genetic variation of the cholesteryl ester transfer protein gene strongly predicts future cardiovascular death in patients with coronary artery disease. J Am Coll Cardiol. 2003;41: 1983–9.

44. Cahilly C, Ballantyne CM, Lim DS, Gotto A, Marian AJ. A variant of p22(phox), involved in generation of reactive oxygen species in the vessel wall, is associated with progression of coronary atherosclerosis. Circ Res. 2000;86: 391–5.

45. Brenner D, Labreuche J, Touboul P-J, Schmidt-Petersen K, Poirier O, Perret C, et al. Cytokine polymorphisms associated with carotid intima-media thickness in stroke patients. Stroke. 2006;37: 1691–6.

46. Hoy A, Leininger-Muller B, Poirier O, Siest G, Gautier M, Elbaz A, et al. Myeloperoxidase polymorphisms in brain infarction. Association with infarct size and functional outcome. Atherosclerosis. 2003;167: 223–30.

47. Pola R, Flex A, Gaetani E, Flore R, Serricchio M, Pola P. Synergistic effect of -174 G/C polymorphism of the interleukin-6 gene promoter and 469 E/K polymorphism of the intercellular adhesion molecule-1 gene in Italian patients with history of ischemic stroke. Stroke. 2003;34: 881–5.

48. Crobu F, Palumbo L, Franco E, Bergerone S, Carturan S, Guarrera S, et al. Role of TGF-beta1 haplotypes in the occurrence of myocardial infarction in young Italian patients. BMC Med Genet. 2008;9: 13.

49. Yoshida T, Kato K, Fujimaki T, Yokoi K, Oguri M, Watanabe S, et al. Association of a polymorphism of the apolipoprotein E gene with chronic kidney disease in Japanese individuals with metabolic syndrome. Genomics. 2009;93: 221–6.

50. Zhang B, Ye S, Herrmann SM, Eriksson P, de Maat M, Evans A, et al. Functional polymorphism in the regulatory region of gelatinase B gene in relation to severity of coronary atherosclerosis. Circulation. 1999;99: 1788–94.

51. Yamada Y, Matsuo H, Segawa T, Watanabe S, Kato K, Hibino T, et al. Assessment of the genetic component of hypertension. Am J Hypertens. 2006;19: 1158–65.
