# Supplementary material for: Association between kidney function and genetic polymorphisms in atherosclerotic and chronic kidney diseases: A cross-sectional study in Japanese male workers
Source: PLoS One. 2017 Oct 10;12(10):e0185476. doi: 10.1371/journal.pone.0185476 (PMC5634546; doi:10.1371/journal.pone.0185476)
Supplement: S2 Table — (DOCX) [file pone.0185476.s002.docx]

**S2 Table** Crude and adjusted logistic regression analysis of association between eGFR and SNPs

|  | **Crude analysis** | | |  | **Adjusted analysis†** | | | |
| --- | --- | --- | --- | --- | --- | --- | --- | --- |
| **rs#** | Coefficient | 95%CI | P value |  | Coefficient | 95%CI | P value | FDR |
| **rs1801133** | -0.29 | (-0.88, 0.30) | 0.34 |  | -0.14 | (-0.69, 0.42) | 0.63 | 0.84 |
| **rs5063** | 0.10 | (-0.89, 1.09) | 0.84 |  | 0.092 | (-0.84, 1.02) | 0.85 | 0.94 |
| **rs1764391** | -0.19 | (-0.93, 0.56) | 0.63 |  | -0.22 | (-0.93, 0.48) | 0.53 | 0.84 |
| **rs1137100** | 0.39 | (-0.30, 1.09) | 0.27 |  | 0.128 | (-0.53, 0.78) | 0.70 | 0.89 |
| **rs267734** | 0.65 | (-0.89, 2.12) | 0.39 |  | 0.74 | (-0.64, 2.13) | 0.29 | 0.83 |
| **rs1417938** | 0.80 | (-0.37, 1.98) | 0.18 |  | 0.76 | (-0.35, 1.87) | 0.18 | 0.72 |
| **rs6131** | 0.52 | (-0.28, 1.32) | 0.20 |  | 0.34 | (-0.41, 1.09) | 0.37 | 0.84 |
| **rs1800872** | -0.17 | (-0.78, 0.44) | 0.59 |  | -0.004 | (-0.58, 0.57) | 0.99 | 1.00 |
| **rs699** | -0.53 | (-1.27, 0.20) | 0.15 |  | -0.38 | (-1.08, 0.31) | 0.28 | 0.83 |
| **rs4762** | 0.14 | (-0.81, 1.08) | 0.78 |  | 0.00 | (-0.89, 0.89) | 1.00 | 1.00 |
| **rs1805087** | -0.27 | (-1.00, 0.46) | 0.46 |  | -0.24 | (-0.93, 0.45) | 0.50 | 0.84 |
| **rs1260326** | -0.28 | (-0.86, 0.30) | 0.35 |  | -0.44 | (-0.99, 0.10) | 0.11 | 0.6 |
| **rs2228048** | 0.010 | (-0.64, 0.67) | 0.97 |  | 0.17 | (-0.45, 0.79) | 0.59 | 0.84 |
| **rs933135** | -0.14 | (-0.79, 0.51) | 0.67 |  | -0.35 | (-0.97, 0.26) | 0.26 | 0.83 |
| **rs3732379** | 1.47 | (0.15, 2.79) | 0.030* |  | 1.25 | (0.004, 2.50) | 0.049* | 0.36 |
| **rs1050450** | 0.43 | (-0.72, 1.59) | 0.46 |  | 0.38 | (-0.71, 1.47) | 0.49 | 0.84 |
| **rs347685** | -0.060 | (-0.68, 0.56) | 0.85 |  | 0.19 | (-0.39, 0.78) | 0.52 | 0.84 |
| **rs388915** | 0.060 | (-0.74, 0.86) | 0.89 |  | -0.023 | (-0.78, 0.73) | 0.95 | 1.00 |
| **rs5186** | 0.050 | (-1.01, 1.12) | 0.92 |  | -0.37 | (-1.37, 0.63) | 0.47 | 0.84 |
| **rs6141** | -0.31 | (-0.89, 0.27) | 0.29 |  | -0.23 | (-0.77, 0.32) | 0.42 | 0.84 |
| **rs4961** | 0.71 | (0.14, 1.28) | 0.015* |  | 0.46 | (-0.076, 1.00) | 0.092 | 0.57 |
| **rs1014290** | 0.15 | (-0.44, 0.74) | 0.61 |  | 0.14 | (-0.42, 0.70) | 0.62 | 0.84 |
| **rs17319721** | -0.89 | (-1.94, 0.17) | 0.099 |  | -1.16 | (-2.15, -0.16) | 0.023* | 0.24 |
| **rs1800591** | 1.00 | (0.22, 1.77) | 0.012* |  | 0.99 | (0.26, 1.72) | 0.0077* | 0.13 |
| **rs1799883** | -0.14 | (-0.74, 0.47) | 0.66 |  | -0.19 | (-0.76, 0.38) | 0.51 | 0.84 |
| **rs1801394** | -0.19 | (-0.81, 0.44) | 0.56 |  | -0.2 | (-0.79, 0.39) | 0.51 | 0.84 |
| **rs11959928** | 0.46 | (-0.23, 1.15) | 0.19 |  | 0.44 | (-0.21, 1.09) | 0.18 | 0.72 |
| **rs2569190** | 0.32 | (-0.27, 0.90) | 0.29 |  | 0.41 | (-0.14, 0.96) | 0.14 | 0.65 |
| **rs2070600** | -0.77 | (-1.58, 0.045) | 0.064 |  | -0.65 | (-1.41, 0.12) | 0.097 | 0.57 |
| **rs881858** | 0.060 | (-0.83, 0.94) | 0.90 |  | -0.13 | (-0.96, 0.71) | 0.77 | 0.91 |
| **rs2431260** | -0.070 | (-0.65, 0.50) | 0.80 |  | 0.012 | (-0.53, 0.55) | 0.97 | 1.00 |
| **rs2070744** | -0.38 | (-1.31, 0.54) | 0.42 |  | -0.26 | (-1.14, 0.61) | 0.55 | 0.84 |
| **rs1799983** | -0.68 | (-1.75, 0.39) | 0.21 |  | -0.75 | (-1.76, 0.26) | 0.14 | 0.65 |
| **rs328** | 0.39 | (-0.48, 1.26) | 0.38 |  | 0.44 | (-0.38, 1.26) | 0.30 | 0.83 |
| **rs10109414** | -0.24 | (-0.96, 0.48) | 0.51 |  | -0.27 | (-0.94, 0.41) | 0.44 | 0.84 |
| **rs1346044** | 0.30 | (-0.79, 1.38) | 0.59 |  | 0.35 | (-0.68, 1.37) | 0.51 | 0.84 |
| **rs4744712** | -0.60 | (-1.19, -0.0080) | 0.047* |  | -0.75 | (-1.31, -0.19) | 0.0088* | 0.13 |
| **rs501120** | 0.04 | (-0.57, 0.64) | 0.90 |  | -0.11 | (-0.68, 0.46) | 0.71 | 0.89 |
| **rs662799** | -0.49 | (-1.09, 0.11) | 0.11 |  | -0.61 | (-1.18, -0.046) | 0.034* | 0.29 |
| **rs5443** | -0.22 | (-0.79, 0.35) | 0.44 |  | -0.13 | (-0.67, 0.41) | 0.64 | 0.84 |
| **rs11053646** | -0.39 | (-1.10, 0.33) | 0.29 |  | -0.24 | (-0.91, 0.43) | 0.48 | 0.84 |
| **rs958812** | -0.24 | (-0.83, 0.35) | 0.43 |  | -0.13 | (-0.69, 0.43) | 0.64 | 0.84 |
| **rs1799986** | 0.40 | (-0.51, 1.32) | 0.39 |  | 0.36 | (-0.50, 1.22) | 0.41 | 0.84 |
| **rs3782886** | -1.74 | (-2.39, -1.09) | <0.0001* |  | -1.63 | (-2.25, -1.02) | <0.0001* | <0.0001** |
| **rs1411766** | -0.020 | (-0.95, 0.91) | 0.97 |  | 0.141 | (-0.74, 1.02) | 0.75 | 0.91 |
| **rs6046** | -0.45 | (-1.63, 0.73) | 0.46 |  | -0.68 | (-1.80, 0.43) | 0.23 | 0.79 |
| **rs2467853** | 2.58 | (1.33, 3.83) | 0.0001* |  | 2.07 | (0.89, 3.24) | 0.0006* | 0.018** |
| **rs1800588** | 0.24 | (-0.34, 0.81) | 0.42 |  | 0.14 | (-0.41, 0.68) | 0.62 | 0.84 |
| **rs6495446** | -0.010 | (-0.77, 0.76) | 0.98 |  | 0.08 | (-0.64, 0.80) | 0.83 | 0.94 |
| **rs5882** | 0.12 | (-0.45, 0.70) | 0.67 |  | 0.084 | (-0.46, 0.62) | 0.76 | 0.91 |
| **rs4673** | 0.060 | (-0.90, 1.03) | 0.90 |  | -0.024 | (-0.93, 0.89) | 0.96 | 1.00 |
| **rs1024611** | -0.79 | (-1.39, -0.19) | 0.0099* |  | -0.65 | (-1.22, -0.084) | 0.025* | 0.24 |
| **rs2333227** | 0.68 | (-0.28, 1.65) | 0.17 |  | 0.44 | (-0.47, 1.35) | 0.34 | 0.84 |
| **rs5498** | 0.19 | (-0.40, 0.78) | 0.53 |  | 0.14 | (-0.42, 0.70) | 0.63 | 0.84 |
| **rs1800469** | 0.39 | (-0.19, 0.97) | 0.18 |  | 0.35 | (-0.20, 0.89) | 0.21 | 0.78 |
| **rs405509** | -0.29 | (-0.93, 0.35) | 0.37 |  | -0.28 | (-0.88, 0.32) | 0.36 | 0.84 |
| **rs13038305** | 0.23 | (-0.62, 1.08) | 0.60 |  | 0.403 | (-0.40, 1.20) | 0.32 | 0.84 |
| **rs3918242** | -0.10 | (-0.89, 0.70) | 0.81 |  | -0.039 | (-0.79, 0.71) | 0.92 | 1.00 |
| **rs5629** | 0.06 | (-0.62, 0.74) | 0.87 |  | -0.078 | (-0.72, 0.56) | 0.81 | 0.94 |

† Adjusted for age, body mass index, fasting blood sugar, and systolic blood pressure

* P < 0.05

** FDR < 0.05
