# Supplementary material for: Association between kidney function and genetic polymorphisms in atherosclerotic and chronic kidney diseases: A cross-sectional study in Japanese male workers
Source: PLoS One. 2017 Oct 10;12(10):e0185476. doi: 10.1371/journal.pone.0185476 (PMC5634546; doi:10.1371/journal.pone.0185476)
Supplement: S3 Table — (DOCX) [file pone.0185476.s003.docx]

**Supplementary Table 3** Result of logistic regression analysis performed for weighting

| rs# | Near gene | Major/minor allele | Risk allele^a^ | Coefficient^b^ | 95% CI | P value |
| --- | --- | --- | --- | --- | --- | --- |
| rs3732379 | *CX3CR1* | C/T | C | -0.27 | (-0.63, 0.094) | 0.15 |
| rs17319721 | *SHROOM3* | G/A | A | -0.094 | (-0.36, 0.17) | 0.49 |
| rs1800591 | *MTP* | G/T | G | -0.27 | (-0.48, -0.07) | 0.009 |
| rs4744712 | *PIP5K1B* | C/A | A | 0.085 | (-0.059, 0.23) | 0.25 |
| rs662799 | *APOA5* | A/G | G | 0.048 | (-0.098, 0.19) | 0.52 |
| rs3782886 | *BRAP* | A/G | G | 0.15 | (-0.0066, 0.31) | 0.061 |
| rs2467853 | *SPATA5L1* | G/T | G | -0.3 | (-0.65, 0.04) | 0.086 |
| rs1024611 | *MCP1* | C/T | T | 0.24 | (0.097, 0.38) | <0.001 |

rs#, rs number; CI, confidence interval; FDR, false discovery rate

^a^ The allele that decreased eGFR was defined as risk allele.

^b^ Coefficient represents logit in logistic regression analysis.
