# Supplementary material for: Association between kidney function and genetic polymorphisms in atherosclerotic and chronic kidney diseases: A cross-sectional study in Japanese male workers
Source: PLoS One. 2017 Oct 10;12(10):e0185476. doi: 10.1371/journal.pone.0185476 (PMC5634546; doi:10.1371/journal.pone.0185476)
Supplement: S4 Table — (DOCX) [file pone.0185476.s004.docx]

**Supplementary Table 4** Univariate and multivariate logistic regression analysis of weighted SNP score and CKD

|  | OR | 95% CI | P value |  | c-statistics GRS (+) | c-statistics GRS (-) | P value |  | IDI | P value |  | NRI | P value |
| --- | --- | --- | --- | --- | --- | --- | --- | --- | --- | --- | --- | --- | --- |
| univariate | 1.05 | [1.03–1.07] | <0.001* |  | 0.574 | - | - |  | - | - |  | - | - |
| model 1 | 1.05 | [1.03–1.07] | <0.001* |  | 0.689 | 0.674 | 0.0086* |  | 0.0073 | <0.001* |  | 0.196 | <0.001* |
| model 2 | 1.05 | [1.03–1.07] | <0.001* |  | 0.718 | 0.709 | 0.036* |  | 0.0064 | <0.001* |  | 0.178 | <0.001* |
| model 3 | 1.05 | [1.03–1.07] | <0.001* |  | 0.781 | 0.775 | 0.040* |  | 0.0076 | <0.001* |  | 0.192 | <0.001* |

CI: confidence interval; GRS: genetic risk score; IDI: integrated discrimination improvement; NRI: net reclassification improvement

Model 1: adjusted for age, body mass index, systolic blood pressure, and fasting blood glucose

Model 2: model 1 + exercise habit, drinking habit, smoking, and stress

Model 3: model 2 + LDL cholesterol and fasting blood glucose, uric acid, and urinary protein through positive urine dipstick

* *P* < 0.05
